# *Conus:* First Comprehensive Conservation Red List Assessment of a Marine Gastropod Mollusc Genus

**DOI:** 10.1371/journal.pone.0083353

**Published:** 2013-12-23

**Authors:** Howard Peters, Bethan C. O'Leary, Julie P. Hawkins, Kent E. Carpenter, Callum M. Roberts

**Affiliations:** 1 Environment Department, University of York, York, United Kingdom; 2 International Union for Conservation of Nature, Global Marine Species Assessment, Biological Sciences, Old Dominion University, Norfolk, Virginia, United States Of America; University of Kent, United Kingdom

## Abstract

Marine molluscs represent an estimated 23% of all extant marine taxa, but research into their conservation status has so far failed to reflect this importance, with minimal inclusion on the authoritative Red List of the International Union for the Conservation of Nature (IUCN). We assessed the status of all 632 valid species of the tropical marine gastropod mollusc, *Conus* (cone snails), using Red List standards and procedures to lay the groundwork for future decadal monitoring, one of the first fully comprehensive global assessments of a marine taxon. Three-quarters (75.6%) of species were not currently considered at risk of extinction owing to their wide distribution and perceived abundance. However, 6.5% were considered threatened with extinction with a further 4.1% near threatened. Data deficiency prevented 13.8% of species from being categorised although they also possess characteristics that signal concern. Where hotspots of endemism occur, most notably in the Eastern Atlantic, 42.9% of the 98 species from that biogeographical region were classified as threatened or near threatened with extinction. All 14 species included in the highest categories of Critically Endangered and Endangered are endemic to either Cape Verde or Senegal, with each of the three Critically Endangered species restricted to single islands in Cape Verde. Threats to all these species are driven by habitat loss and anthropogenic disturbance, in particular from urban pollution, tourism and coastal development. Our findings show that levels of extinction risk to which cone snails are exposed are of a similar magnitude to those seen in many fully assessed terrestrial taxa. The widely held view that marine species are less at risk is not upheld.

## Introduction

Extinction risk of marine organisms has attracted little attention compared to that of terrestrial taxa, with a widely held view that such risk is inconsequential due to high dispersal ability and large geographic ranges [1], [2] especially when taking reference from the fossil record [1], [3]. These beliefs are particularly prevalent when considering marine invertebrates, where a decline in abundance of the important phylum *Mollusca* has been overshadowed by the collapse in many exploited vertebrates, especially finfish [4]. This is primarily due to their relatively minor contribution to human protein requirements and the generally held belief that molluscs possess greater resilience to extinction through their perceived wide distribution and a likelihood of hidden pockets of survivors [5]. Marine invertebrates in general are seriously under-represented within the IUCN Red List [6]. Only cuttlefish, lobsters and scleractinian corals have been fully assessed and published [6], [7]. Although limited research on the impact of habitat loss and fishing pressure on marine gastropod molluscs has been undertaken on a regional scale including for shell fisheries [8], [9], there have been no comprehensive assessments of trends in species abundance, commercial and environmental impacts and extinction risk to any genera with a global biogeographical distribution.

Cone snails of the genus *Conus* offer an excellent opportunity to explore global threats to marine molluscs owing to their exceptional diversity [10], wide distribution, high degree of endemism, varied depth distribution [11], and an established global market in their trade from amateur shell collectors to commercial traders [12]. In addition, cone snails are used in some communities in the Pacific as an occasional foodstuff [13] but, more importantly, they are actively targeted by international drug companies and researchers as a potential pharmacological resource [14].

Cone snails constitute the family *Conidae*, which together with the *Turridae* (turrid snails) and *Terebridae* (auger snails) comprise the superfamily *Conoidea* otherwise known as *Toxoglossa* (‘poison tongue’) owing to the venom apparatus they deploy for immobilising prey [15]. The *Conoidea* form part of the order *Neogastropoda* in the sub-class *Prosobranchia* of the class *Gastropoda* of the phylum *Mollusca* [11].

Cone snails live throughout the world's tropical coastal waters with a steep latitudinal diversity gradient away from the tropics, extending into cooler regions that include southern California, northern Gulf of Mexico, Florida and the Carolinas, North Africa, the Mediterranean, South Africa, Australia, southern Japan and China [16]. Distribution varies widely with some species occurring across the entire tropical Indo-Pacific but others restricted to a single bay or seamount [11], [17].

The genus *Conus* is taxonomically challenging. Although morphological characteristics of the shell remain the initial means of species identification [11], more recently, other traits have also been employed to differentiate among species, in particular the radular teeth used in the capture of prey, whose shape and structure not only reflects the dietary preferences of the species [18] but may also be specific to a single species [19]. Separation of species through DNA sequence variations provides even greater reliability, but more recently, character-based DNA barcoding has been highly effective in distinguishing among closely-related species [20]. For this assessment we relied upon expertise from taxonomists in *Conus* to create a dataset of valid species.

The fossil record indicates that the first *Conus* appeared in a sea that covered what is now England and France during the Lower Eocene around 55 mya [21]. During subsequent radiations the genus expanded around the globe and by the Holocene had formed into four biogeographical regions: Indo-Pacific (IP), Eastern Pacific (EP), Western Atlantic (WA), and Eastern Atlantic (EA). Although widening of the Atlantic during the Cretaceous and Cenezoic has today created an impermeable barrier to *Conus* crossing the ocean, there have been some migrations in the past, as witnessed from the fossil record and more recently by *C. ermineus* extant in both the EA and WA and *C. chaldaeus, C. ebraeus* and *C. tessulatus* found in both the IP and EP [22].

The majority of the 632 species of *Conus* assessed (53.6%) occurs in the infralittoral zone of 5 m deep or less, with most of the remaining species (27.7%) at 50 m deep or less. However, there are some species such as *C. teramachii* that live in deeper parts of the continental shelf extending to 1,000 m where they may be brought to the surface as bycatch of demersal fisheries. The bathymetric ranges of individual species vary considerably with some shallow water species living within a one or two metre depth range and some deep-water species being found within a 500 m range or more [11].

Microhabitats vary by species and most often consist of sand or mud into which the cone snail may burrow, but may also include inter-tidal limestone benches (the smooth remains of reef structures from earlier geological periods when sea levels were higher [23]) with sand or algal turf, sub-tidal reef platforms with living and dead corals, or boulders with sandy layers [24]. They may also be found among coral rubble and occasionally among mangroves and seagrasses.

**Figure 1:**
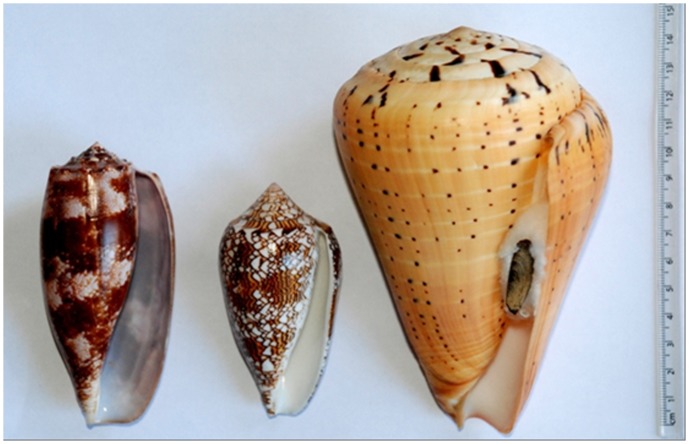
Diet and toxicity. Left: *C. geographus* Linnaeus, 1758; piscivorous, 65–165 mm; intertidal to 20 m; significant fatality risk to humans. Centre: *C. textile* Linnaeus, 1758; molluscivorous, 40–150 mm; intertidal to 50 m; handle with extreme caution. Right: *C. betulinus* Linnaeus, 1758; vermivorous, 55–177 mm; intertidal to 20 m; minimal risk to humans; note operculum. All species Indo-Pacific.

Cone snails are generally nocturnal in their feeding habit [25] and group-specific in their preference for worms, molluscs or fish (Fig. 1) although some species have a mixed diet [26]. The smallest groupings by diet are the obligate piscivores with around 50 species [11], [27], and obligate molluscivores with approximately 80 [28]. The majority of *Conus* are vermivorous with polychaetes representing the largest dietary component, that can be the exclusive source of food for some species [11]. All cone snails use venom to immobilize their prey. The diversity of venoms employed by a particular species in the capture of prey is a reflection of the degree of specialisation in its diet [29].

From the earliest civilizations, people have prized cone shells for their exceptional beauty, with examples discovered among prehistoric artefacts used for personal adornment extending back 5,000 years [30]. Their striking patterns and wide range of colours and shades continue to attract collectors today with rare examples in perfect condition changing hands for thousands of dollars with common and abundant species traded for cents to a dollar or two each [12].

Over millions of years *Conus* has evolved a battery of peptide toxins (conopeptides/conotoxins) for immobilizing prey [31]. The venom of each species is a cocktail mixed from between 50 and 200 different peptides each of only 10 to 35 amino acids in length and is generally targeted at voltage-gated or ligand-gated ion channels [30]. These conopeptides have become a focus for biomedical research worldwide [14]. Indeed, with the probability that there are on average over 100 distinct toxins for each species [30], as a whole, the *Conidae* can probably synthesize in excess of 50,000 toxins with little, if any, replication [32].

Cone snails are therefore important to: a) biodiversity; they have evolved into one of the largest of all marine genera, b) biopharmaceutics; they offer unparalleled opportunities in the development of novel drugs, and c) economics; their shells provide income to poor fishing communities through sales to tourists, traders and a global business in the specimen shell trade.

Habitat loss is considered by many malacologists to be the primary risk factor facing tropical marine mollusc species (Bouchet pers. comm. 2011) and there is plenty of hard evidence to support this view. In Queensland, Australia, for example, abundance and species richness of mollusc assemblages have been shown to be adversely affected by removal of subtropical mangrove forests, with population declines of 83% recorded [33]. In San Diego, Southern California the endemic horn snail, *Cerithidia fuscata*, that lived along intertidal mudflats was last seen in 1935 after pollution and dredging had driven it to extinction [34]. Where coral cover has been extensively damaged or degraded through pollution, sedimentation, coastal development and destructive fishing, as witnessed throughout much of the tropics, coral-associated molluscs such as the *Conidae* are being usurped by bivalve crevice dwellers [35].

In this paper we report one of the first comprehensive extinction risk assessments of a taxonomically well-resolved marine taxon. Our research assesses the extinction risk to the global populations of *Conus*, examining each species' distribution, current and projected threats from disturbance to habitats, pollution, coastal development, and shell gathering. We have examined where possible the effects of fragmentation of populations and the likely impact of demersal fisheries on deeper water species. The assessment enables us to reappraise whether marine taxa are less extinction prone than terrestrial. In addition, our aim is to provide data in support of conservation measures for those species at the greatest risk of extinction over the short to medium term.

## Methods

### Red List Assessment

We used the assessment standards and procedures of the International Union for the Conservation of Nature (IUCN) Red List of Threatened Species to assess extinction risk to 632 species of *Conus*. This is the world's leading resource for describing the global conservation status of plants and animals and uses a standard methodology to classify species into one of nine categories, together with a codified set of criteria [36]. The assessment includes examination of the effects of both ecological change and commercial exploitation on the subject taxa. Data derived during the research and discovery process for each species is compiled to a standard format together with maps, images and other supporting documentation.

Following taxonomic review, we divided valid species into 12 biogeographical working sets for detailed assessment. A comprehensive assessment was not possible for those species where data was substantially absent. For example, species endemic to areas of protracted civil unrest such as the Horn of Africa may not have been researched in the field for many years. Coincidentally, these regions are not generally subject to intensive coastal development, harbour works and refineries and so may offer a degree of protection to marine taxa. Similarly, species occurring in deep water, where recovery is most commonly through fisheries by-catch, often suffer a paucity of data including extent of distribution and habitat types. Furthermore, bathymetry data will often rely on the questionable estimation of fishers. Wherever possible for deep-water *Conus*, we have focussed our attention on the level of demersal fishing in the area, including destructive methods such as dredging that may seriously affect mollusc assemblages.

Most *Conus* species, however, occur in shallow water where impacts such as coastal development, pollution and habitat destruction can be more easily recorded. Such threats can give rise to population fragmentation leading to a serious decline in abundance which may be difficult to quantify until it has become extreme. However, indicators including market prices for specimen shells provide a useful guide to increasing scarcity. Knowledge voids are common for *Conus* but where they occur we have, where possible, used estimation or inference using suboptimal data permitted under Red List standards [36]. Despite this, 13.8% of *Conus* species were found to be so deficient in data we were unable to make an assessment with any degree of reliability.

### Key indicators of risk

#### Distribution

A key indicator of potential risk to a species is the size of its geographical distribution. The Red List standard assessment uses two measures: Extent of Occurrence (EOO) and Area of Occupancy (AOO). EOO for marine species is the area within a polygon drawn around the boundary of the species' range, excluding land areas. This will include areas which may not be physically occupied by the taxon, e.g. deep water, but which could contribute to larval dispersal. AOO is the physical area within the EOO in which the taxon is known to occur. For shallow water species, this may be calculated from the perimeter of an island or length of coastline, extended by the width of habitat calculated from the known or inferred bathymetric range of the species over the area under review. However, for ‘linear’ habitats such as rivers and coastlines, IUCN suggests that their standard habitat width of 2 km should be used in computing AOO [36] and we have adopted this approach in the assessment for *Conus*. It should be noted that both the AOO and the EOO are only of major significance in assessing the level of threat if the species has a restricted range, and that for most wide-ranging *Conus* species that fall outside the parameters of the assessment criteria these range sizes are not calculated. However, spatial data, derived from range maps created for every species assessment, were projected using ArcGIS version 10.1 (Environmental Systems Research Institute) to generate species richness maps for a) all species and b) only those species with a range less than the median mapped range size. All data were standardised onto 1° grid cells and projected to world cylindrical equal area.

#### Number of Locations

It is possible that a catastrophic event could have a profound effect on the population size of some species. Although marine molluscs are resilient in being able to endure physical forces such as extreme weather events, small populations may be extirpated as a result of sudden habitat loss caused by catastrophic events such as major oil spills. The ‘location’ count indicates the number of areas in which a single catastrophic event could affect all individuals of the taxon present, events that may cumulatively drive a species into extinction. The value of this measure is another key factor in determining the level of risk a global population faces.

### Literature search

We conducted a comprehensive search through published papers and other literature for data relating to *Conus* species' populations, depth, distribution, habitats, trade in animals and shells, use for foodstuff, pharmaceuticals, etc., together with any conservation measures in place, including indirect conservation as may be offered by marine protected areas. We sought information on current and possible future threats, including coastal development for tourism, industry or port construction, nutrient loading from agricultural run-off, pollution from domestic and industrial effluent, intensive trawling, siltation from land-based sources, dredging for shipping channels and mineral extraction. Data on activities such as these can often only be found in trade publications, contract award notifications etc.

We also examined the market in shells to determine ‘collectability’, pricing fluctuations, scarcity and demand. Some shells with exceptional colour and form will achieve iconic status, and if they are also rare like *C. gloriamaris* or *C. milneedwardsi*, it adds to their cachet. Species that live within a highly restricted range, within a single bay for example, are often at heightened risk from human activity. This particularly applies to shallow water species which may be gathered as curios in areas where new beach tourism projects are being developed or planned. We synthesised distribution data including observed fragmentation, location counts, marketability, population declines and threats for each species to apply one of the nine categories listed below.

### Assessment categories

There are three categories of extinction risk: critically Endangered (CR), Endangered (EN) and Vulnerable (VU) that broadly define ‘extremely high’, ‘very high, or ‘high’ risk of extinction respectively. In addition, there are two extinct categories, Extinct (EX) and Extinct in the Wild (EW), and three other categories: Near Threatened (NT) for species that will be elevated to a threatened category in the short term unless the potential risk is removed; Data Deficient (DD) where there is insufficient data to determine a category, and Least Concern (LC) where current and projected population levels indicate the species is not at risk. As this was a comprehensive assessment, we did not use the category Not Evaluated (NE), where the species has been recorded but no assessment has been carried out.

For *Conus*, the criteria in support of the selected category are primarily derived from a range of variables based on estimated population size and/or level of decline together with species range size and location count.

### Synthesis and pre-publication checks

Following our research and assessment, the results were reviewed by a panel of fourteen international experts, each with specialist knowledge of the *Conus* species within their allotted biogeographical working sets. The review took the form of a five day synthesis workshop with teams comprising leading academics together with renowned specialists from the commercial sector with comprehensive field knowledge of species' distribution, scarcity and threats, and a facilitator experienced in Red List standards and procedures. This peer-review process confirmed or modified findings of the original assessment authors, and allowed inclusion of supplementary field-based knowledge from the participating experts. All reports were checked for consistency by the Mollusc Specialist Group of the IUCN Species Survival Commission before final approval and submission for publication through the IUCN Red List Unit.

## Results

**Figure 2:**
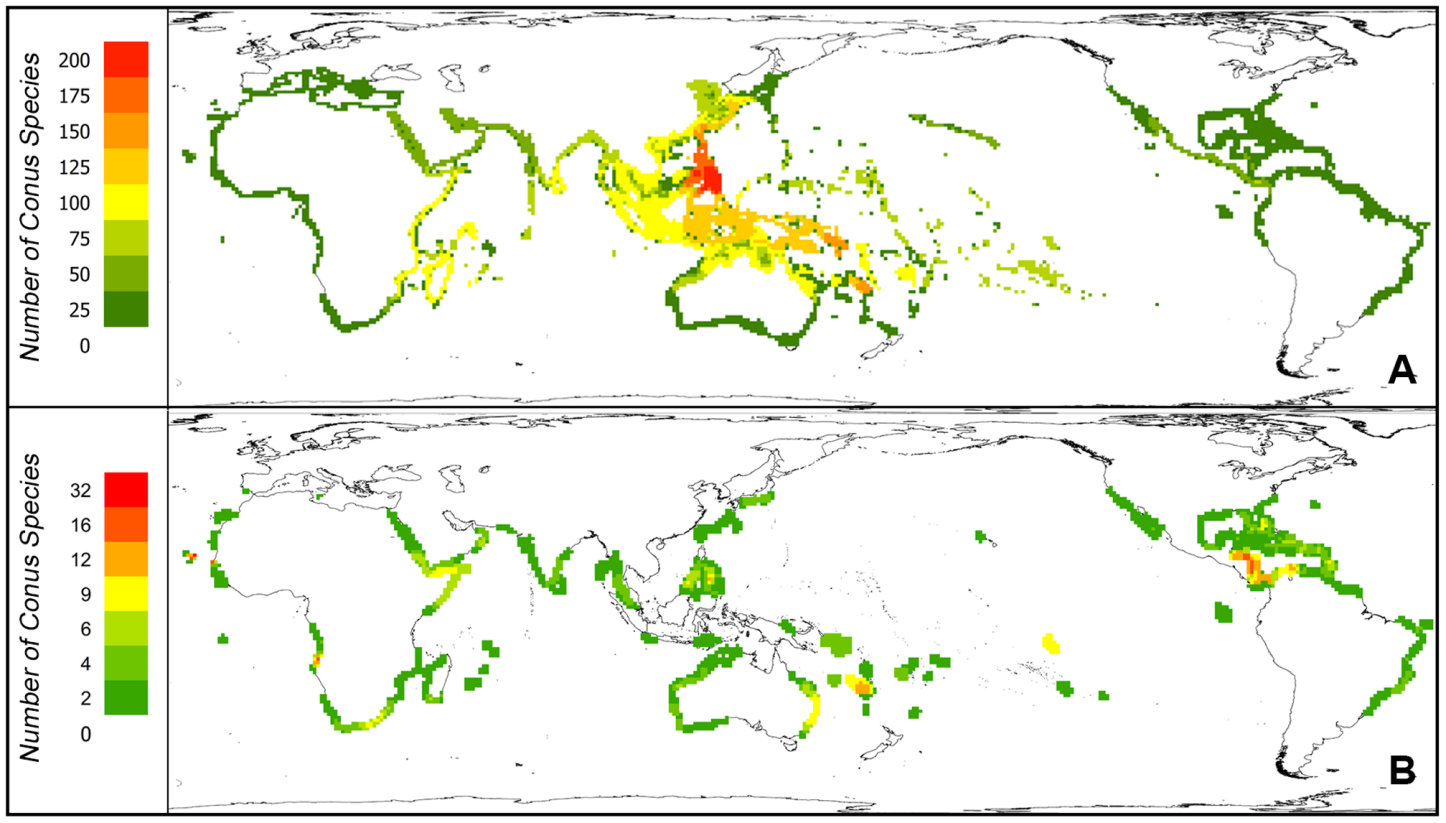
*Conus* species distribution. Species richness from a composite of individual species maps (Fig. 2A), and only species with mapped area less than the median indicating regions of potential endemism (Fig. 2B).

The greatest species richness for *Conus* is in the Philippines and countries to the south and east towards Papua New Guinea, the Solomon Islands, New Caledonia and Fiji (Fig. 2A). Species occurring in the Atlantic including West Africa and the Gulf of Mexico and Caribbean are fewer in number. However, when very wide-ranging species are omitted, i.e. those equal to or greater than the median mapped range size, centres of endemism are revealed in scattered locations including the Caribbean, the Marquesas, New Caledonia and the Eastern Atlantic, in particular Cape Verde, Senegal and Angola (Fig. 2B).

### Global threats

**Figure 3:**
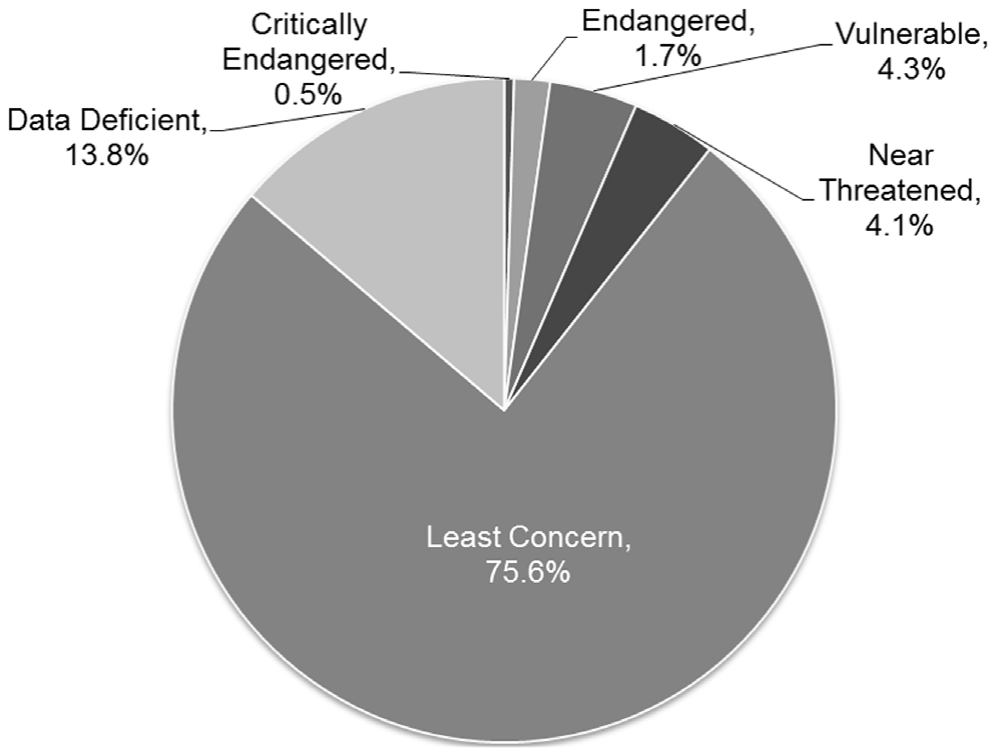
Global extinction risk to *Conus*. The percentage contribution for each assessed category to the global diversity of 632 *spp* of *Conus*. These are represented by 3 Critically Endangered species; 11 Endangered; 27 Vulnerable; 26 Near Threatened; 87 Data Deficient, and 478 of Least Concern.

Three of 632 *Conus* species assessed were considered to be Critically Endangered (CR), 11 Endangered (EN) and 27 Vulnerable (VU), which together represent 6.5% of all global species (Fig. 3), with a further 26 species (4.1%) categorised as Near Threatened (NT). Over one in ten of all *Conus* species is therefore considered at risk or may become so in the near future. 87 species (13.8%) were categorised as Data Deficient (DD) of which 75 (86.2%) occur in the Indo-Pacific.

**Figure 4:**
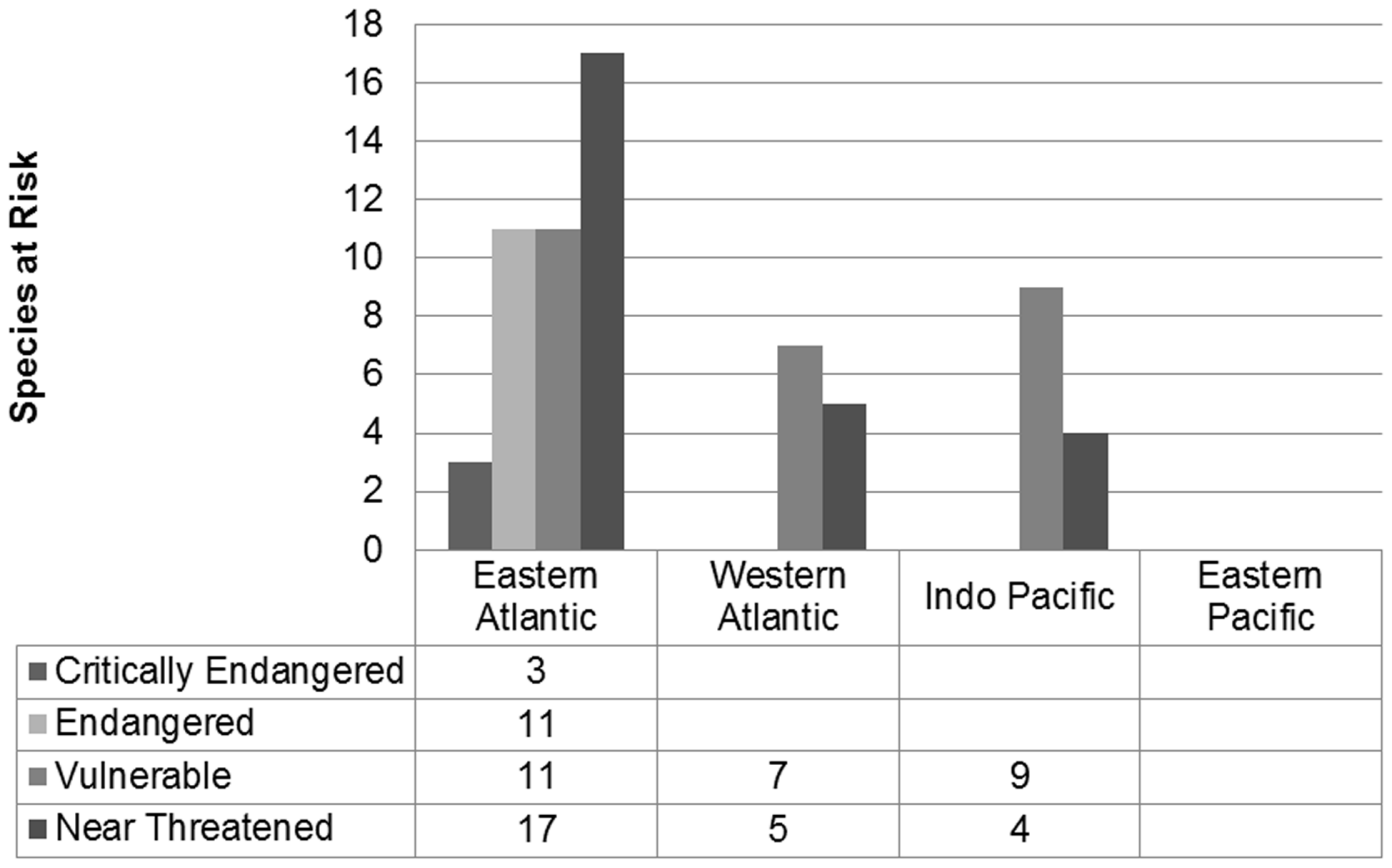
Number of *Conus* species at risk by ocean basin. The number of species at risk by ocean basin for each threatened category. There are no species at risk in the Eastern Pacific.

All 14 CR and EN species occur in the waters off Cape Verde and Senegal, West Africa (Table 1). Of the 27 assessed as VU, eight are from Cape Verde and Senegal with three from Angola (Table 1), seven from the Western Atlantic (Table 2), and nine from the Indian Ocean, including two from Western Australia (Table 3). Only three threatened species occur east of longitude 60 (Oman to Mascarenes): *C. rawaiensis* from Western Thailand and *C. compressus* and *C. thevenardensis* from Western Australia – all VU. According to this assessment procedure, there are no threatened species in the Pacific (Fig. 4). Of the 26 Near-Threatened species (NT), Cape Verde and Senegal are again over-represented with 14 of the 17 species from the Eastern Atlantic. Of the remainder in this category, five are from the Western Atlantic, one from the Western Indian Ocean, and three from the Pacific (Fig. 4).

**Table 1:** Threatened *Conus* of the Eastern Atlantic (EA).

Critically Endangered (CR)		Endangered (EN)		Vulnerable (VU)	
Cape Verde	*C. lugubris*	Cape Verde	*C. ateralbus*	Angola	*C. allaryi*
Cape Verde	*C. mordeirae*	Cape Verde	*C. crotchii*	Angola	*C. cepasi*
Cape Verde	*C. salreiensis*	Cape Verde	*C. cuneolus*	Angola	*C. xicoi*
		Cape Verde	*C. fernandesi*	Cape Verde	*C. decoratus*
		Senegal	*C. belairensis*	Cape Verde	*C. felitae*
		Senegal	*C. bruguieresi*	Cape Verde	*C. fontonae*
		Senegal	*C. cloveri*	Cape Verde	*C. regonae*
		Senegal	*C. echinophilus*	Cape Verde	*C. teodorae*
		Senegal	*C. hybridus*	Senegal	*C. cacao*
		Senegal	*C. mercator*	Senegal	*C. guinaicus*
		Senegal	*C. unifasciatus*	Senegal	*C. tacomae*

**Table 2:** Threatened *Conus* of the Western Atlantic (WA).

Vulnerable (VU)	
Aruba	*C. hieroglyphus*
Florida	*C. anabathrum*
Florida	*C. stearnsii*
Bahamas	*C. richardbinghami*
Brazil	*C. henckesi*
Martinique	*C. hennequini*
Venezuela	*C. duffyi*

**Table 3:** Threatened *Conus* of the Indo-Pacific (IP).

Vulnerable (VU)	
Oman	*C. ardisiaceus*
Oman	*C. melvilli*
S Red Sea	*C. cuvieri*
Mascarenes	*C. julii*
Réunion	*C. jeanmartini*
SE South Africa	*C. immelmani*
W Thailand	*C. rawaiensis*
Australia	*C. compressus*
Australia	*C. thevenardensis*

### Analysis by Region

**Figure 5:**
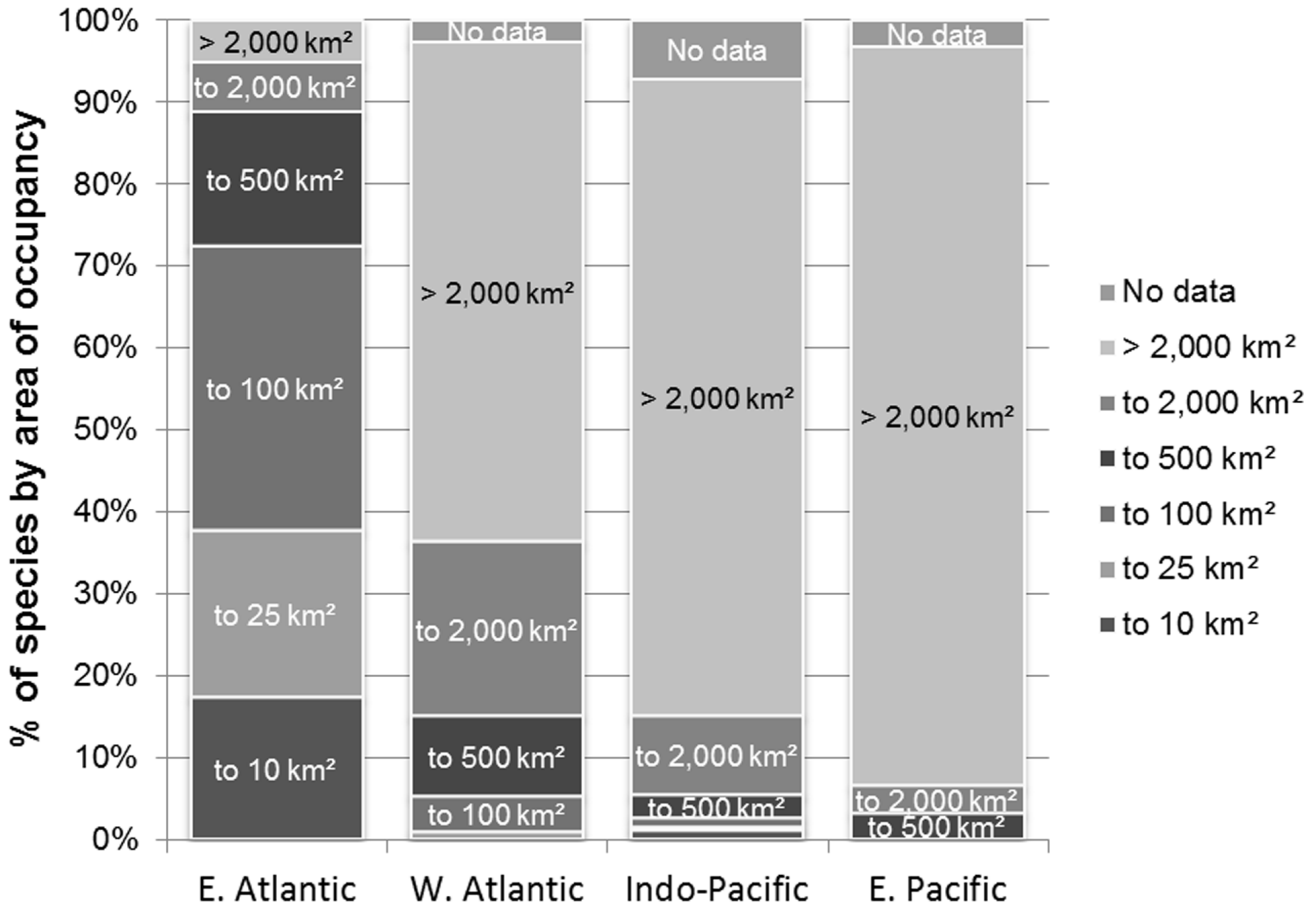
Contribution of range-restricted species to *Conus* biodiversity within each ocean basin. This illustrates by region the percentage of total species by area of occupancy, with wide-ranging species, i.e. >2,000 km^2^ being minimal in the Eastern Atlantic but the major contributor to the Indo-Pacific and Eastern Pacific *Conus*. The abbreviated key describes the band sizes, e.g. to 10 km^2^ = 0–10 km^2^, to 25 km^2^ = 11–25 km^2^, to 100 km^2^ = 26–100 km^2^, etc.

Marine molluscs that are wide-ranging are likely to be more resilient against threats than those that are range-restricted, with dispersed populations providing a reservoir for re-colonization in the event of local extirpations [2]. The Eastern Atlantic species occupy a limited length of coast with few islands when compared to the Western Atlantic and, more particularly, the Indo-Pacific. It is also intersected by large rivers draining the tropical land mass of Africa which render substantial areas of coastal water unsuitable for many marine molluscs. Conversely, islands of the tropical Indo-Pacific and Caribbean contribute substantial areas of shallow water habitat suitable for taxa such as *Conus* and do not generally suffer any significant flux of freshwater. Figure 5 shows the percentage distribution of species' range sizes within each of the four oceanic regions. This graphically illustrates that wide-ranging *Conus* species, i.e. AOO >2,000 km^2^, are uncommon within the Eastern Atlantic compared to the other regions.

#### Eastern Atlantic

Ninety-eight species of *Conus* occur along the Eastern Atlantic seaboard from the Mediterranean and Morocco south to Namibia, with associated island archipelagos including the Canaries, Azores, and Cape Verde (plus one: *C. ermineus*, that also occurs in the Western Atlantic and was included in that region). There is one species from the island of St Helena, *C jourdani*, within this grouping although no live specimens have been observed and it is categorised as DD. With three CR, 11 EN and 11 VU species, representing 25.5% of the Eastern Atlantic species, and a further 17 species NT (Table 1), 42.9% of Eastern Atlantic *Conus* are considered at risk of extinction or liable to become so. This exceptional concentration of threatened species is found nowhere else across the genus' wide distribution and the disproportionate contribution of species from Cape Verde and Senegal demands further explanation.

Cape Verde is home to 8.9% of all *Conus* species. With 53 species endemic from a total of 56 present in the archipelago, endemism is exceptionally high at 94.6%. Forty-three species are each restricted to a single island. All three CR species are found in Cape Verde, *C. lugubris, C. mordeirae*, and *C. salreiensis*, together with four EN and five VU (Table 1). There are also 12 NT species. With 24 species in either a threatened or near-threatened category, Cape Verde has 45.3% of its *Conus* diversity at risk compared to 7.4% for the remainder of the world. Angola and Senegal contribute the next largest numbers of endemic *Conus* species with 22 and 13 respectively, which together with 53 species endemic to Cape Verde account for 89.8% of all 98 species within the Eastern Atlantic. Senegal contributes seven EN and three VU species with Angola contributing three VU (Table 1).

#### Western Atlantic

We assessed 113 species of *Conus* from the Western Atlantic where they occur from the Carolinas and Bermuda south to Brazil and throughout the Gulf of Mexico and Caribbean. Species are widely variable in their distribution across the region. There are six threatened species, all categorised as VU (see Table 2), representing 5.3% of the total and a further four NT, together resulting in 8.8% of *Conus* species within this region considered at immediate or potential risk.

#### Indo-Pacific

We assessed 390 species of *Conus* from the Indo-Pacific where they occur across the tropics and subtropics, from East Africa south to South Africa and north to the Red Sea and the Persian Gulf and across the whole of the Indian Ocean and the Western and Central Pacific, south to Australia and New Zealand, north to Japan, east to French Polynesia and Easter Island and northeast to Hawaii.

Only nine species were found to be VU. All occur within the Indian Ocean with six species from the western flank: two from Oman, one from the southern Red Sea, two from the Mascarenes and one from South Africa. From the eastern flank there is one species from Thailand and two from Western Australia. There are also four NT species including one from Oman with the other three being the only *Conus* species potentially at risk in the Pacific – one each from Queensland, the Philippines and the Marquesas.

#### Eastern Pacific

We assessed 31 species of *Conus* from the Eastern Pacific where they occur from Southern California south along the Pacific coast of Meso-America to Southern Ecuador including the Galapagos and other island groups of the region.

No species were assessed as threatened or near threatened in the Eastern Pacific.

### Threats

The nature of threats to those species of *Conus* at risk of extinction are varied and depend primarily, but not exclusively, on the proximity and nature of human habitation and development adjacent to coastlines where the molluscs occur. This alone, however, will not normally create a scenario for species extinction. Wide-ranging species are capable of maintaining their viability through resilience from multiple sub-populations. Although most threatened *Conus* species are range-restricted, this is not always the case: two species from the USA, *C. anabathrum* and *C. stearnsii* occur along the west coast of Florida where their ranges are substantially fragmented by shoreline development. However, restricted range, coupled with shallow water habitat, magnifies the impact of stressors such as coastal development or pollution. Of the 41 *Conus* species globally assessed as threatened with extinction, 32 (78.0%) occur within an AOO of 250 km^2^ and a minimum depth of 5 m or less. In the Eastern Atlantic, of the 25 threatened species, this rises to 100%.

**Figure 6:**
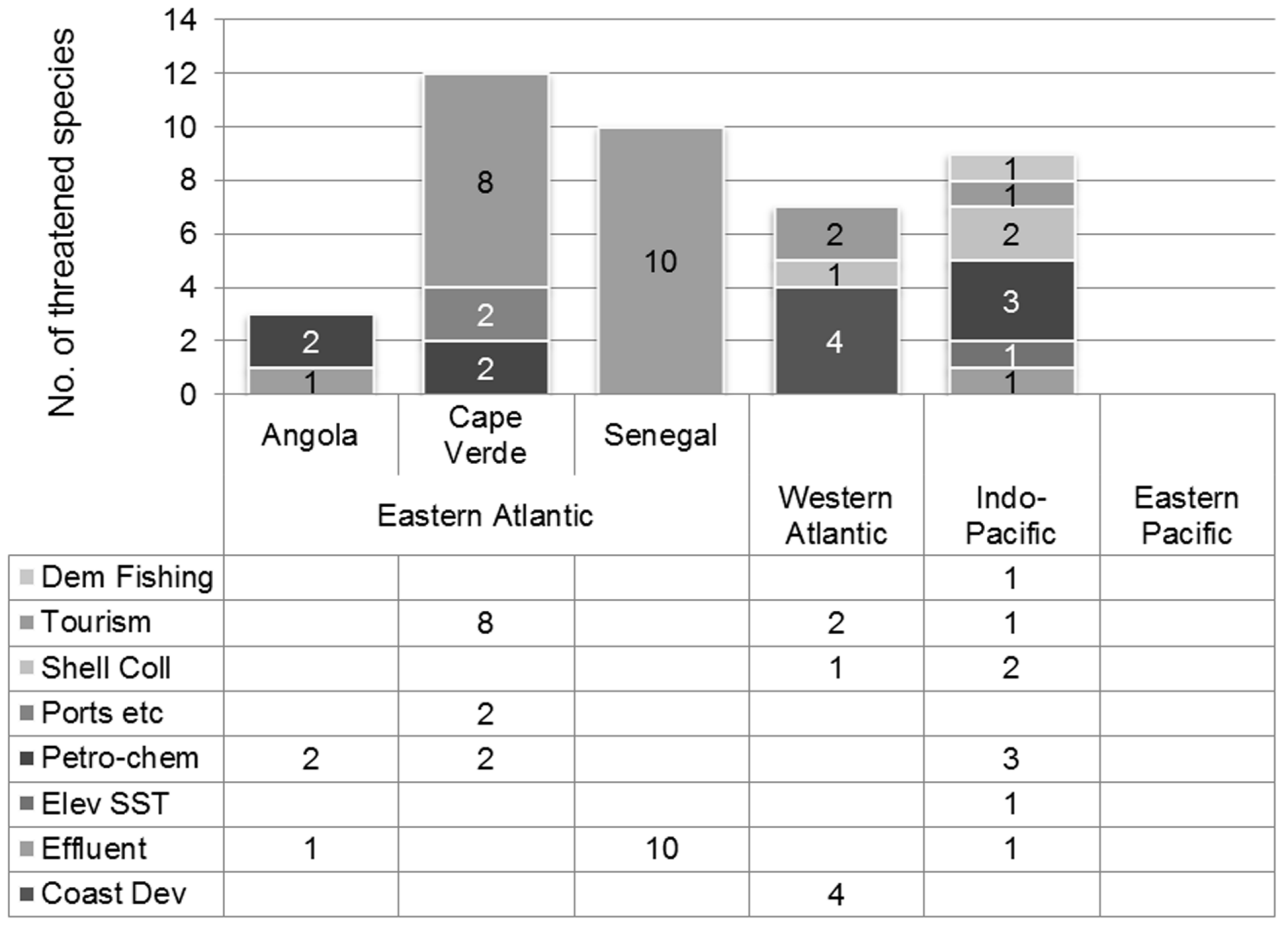
Main threats to *Conus* by ocean basin. The number of *Conus* species at risk (consolidation of CR, EN and VU) indicating primary causes of endangerment, being demersal fishing, tourism, shell collecting, ports and harbours, petro-chemical spills, elevated sea-surface temperatures, effluent discharge and runoff, and coastal development.

Threats to those *Conus* species assessed within one of the three threatened categories can be classified into four causal groups: 1. pollution, either from proximity to actual or potential petro-chemical spills, or urban and industrial effluent; 2. disturbance to habitat from coastal development either resulting from human population increases, e.g. sea defences, residential and commercial structures, including aquaculture facilities, and port construction, or tourism infrastructure. Also included in this group is damage to habitat caused by damaging and extensive demersal fishing; 3. shell gathering, and 4. environmental change e.g. elevated sea-surface temperatures (Fig. 6). There will frequently be a combination of causes, for example tourism infrastructure may also increase shell gathering. Similarly, the proximity of shanty towns devoid of planning regulations poses an elevated risk of effluent discharge into the marine environment. Finally habitat destruction from sand removal, beach nourishment works and recreational use of the sea may all result in disturbance to local mollusc populations.

Cape Verde is experiencing a major structural change from a largely services and fisheries based economy supported by development aid and remittances from its diaspora to one of beach tourism [37]. This is accompanied by a myriad of threats from road and resort construction, unlawful removal of beach sand for cement [38] and casual shell gathering by tourists. All three CR species occur in Cape Verde where their populations are already reduced. *C. lugubris* and *C. mordeirae* live in areas where habitat has already been lost to development and *C. salreiensis* which is restricted to a single bay has had observable declines in population since a harbour was constructed. Each is found in an area along a shallow coastal strip of less than 11 km in length. Harbour expansion and the accidental discharge of engine fuel increase the pressures on small, range-restricted *Conus* populations such as *C. fernandesi, C. fontonae* and *C. regonae*. With so many *Conus* species occupying highly restricted ranges within the archipelago, modest threats such as these could have a profound impact.

Around the Dakar peninsula, Senegal, it has been observed that species restricted to its highly polluted coastal waters are showing a marked decline in abundance coupled with an overall diminution of shell size including *C. echinophilus, C. hybridus, C. mercator* and *C. unifasciatus*. In common with many maritime cities in developing countries, Dakar suffers from a burgeoning population with largely inadequate waste-processing infrastructure. South in Angola, *Conus* species categorised as at risk face similar threats to those in Senegal.

In the Western Atlantic some disturbance to *Conus* can be traced to human migration to the Florida coast. Tourism and retirement have driven large-scale construction projects for condominiums and other coastal infrastructure leading to significant loss of habitat for *C. anabathrum* and *C. stearnsii*. Tourism also represents the underlying risk to the vulnerable species *C. hennequini* in Martinique and *C. hieroglyphus* in Aruba. Shell collecting in the Bahamas threaten *C. richardbinghami*. General coastal development in Bahia, Brazil threatens *C. henckesi* where it occurs only off two small islands. The Venezuelan government has voiced plans for substantial development on the islands of Los Roques which will place the shallow water species, *C. duffyi* at risk.

The *Conus* species of the Indo-Pacific are at less risk. In the north-western Indian Ocean, the Persian Gulf, the southern Red Sea including the Gulf of Aden and the Horn of Africa, civil wars, poverty, piracy and the security situation offer some degree of protection from coastal development. However, there are still concerns from oil spillage in the region and two scarce species from Oman, *C. ardisiaceus* and *C. melvilli* together with *C. cuvieri* from Djibouti are categorised as VU. In Southern Natal and the Mascarene islands of Mauritius and Réunion respectively, *C. immelmani* and *C. julii*, have both declined in numbers almost certainly from over-collecting, with *C. jeanmartini* also from Réunion being subject to intensive trawling in its deep-water habitat.

In the Eastern Indian Ocean, *C. rawaiensis* occurs only in an area estimated at less than 35 km^2^ in a single location off the western shores of Thailand in a region zoned for tourism. In Western Australia, an extreme localized warm-water event in 2011 from La Niña, in the region around Geraldton to Shark Bay including the Abrolhos Islands, resulted in a catastrophic decline of marine molluscs including *Conidae*. *C. compressus*, a restricted range species, possibly suffered a 50% decline in abundance. Also in Western Australia, *C. thevenardensis*, already rare, is subject to a range of threats including a large oil installation, tourism and dredging.

### Other Red List categories

The results for the three threatened categories paint an incomplete picture. There are also 87 species assessed to be Data Deficient and 26 as Near Threatened, together representing 17.9% of the global diversity. Many of the Data Deficient species are considered to be scarce in the wild even though the causes and extent of the threats they face cannot yet be determined with sufficient accuracy. Twice as many of the 87 DD species, (39.1%) occur only at depths of 50 m or more, i.e. below gleaning, scuba, and (many) artisanal fishing gears, compared to 19.4% for the remaining 545 species globally. Specimens may be brought to the surface from these depths as by-catch from fisheries. However, demersal gear such as dredges may also contribute substantially to the endangerment of the species recovered through destruction of their habitat, especially for those that are also of restricted range. Occurrence in deep water does not automatically result in a DD categorisation. Despite paucity of data, taxa with a known distribution greater than 2,000 km^2^ but with no known threat would normally be assessed as Least Concern. At the other extreme, there are also a number of DD species where there is an almost total absence of recent sightings but extinction cannot be proven, i.e. there is reasonable doubt that the last individual has died [39]. This is exemplified by species such as *C. jourdani* from St Helena which is only known from ‘dead’ shells washed onto the beach in one small bay; *C. bellulus* and *C. luteus* which have not been reported since the 1970s; *C. splendidulus* which has not been seen in 20 years and *C. sauros* which is possibly extinct.

**Figure 7:**
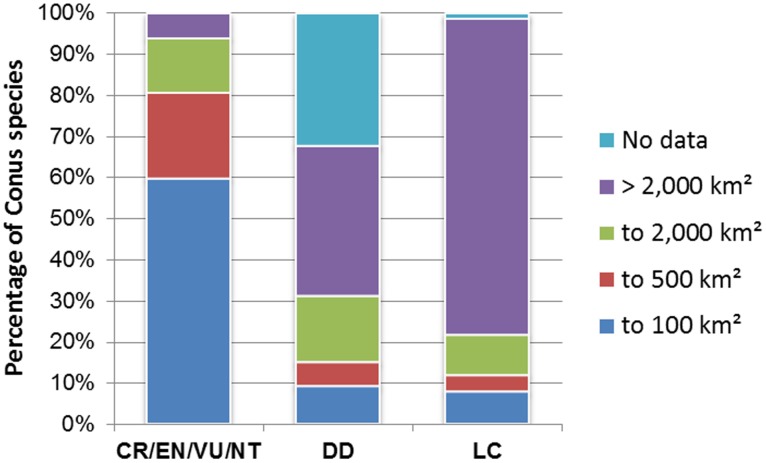
Contribution of range-restricted *Conus* species within assessment categories. This shows the percentage of species by assessment category, with DD species having a higher proportion of taxa with small ranges compared to LC species and also with a large number of species with no distribution data. All threatened and near-threatened categories have been grouped. Key: CR Critically Endangered, EN Endangered, VU Vulnerable, NT Near Threatened, DD Data Deficient and LC Least Concern

Threatened species frequently have small ranges. Of 478 *Conus* species categorised as LC, 103 have an AOO of 2,000 km^2^ of less (21.5%). However, of the 67 species with threatened and near-threatened categories, all except four occur within an AOO of 2,000 km^2^ or less (94.0%), with 40 (59.7%) restricted to a range of 100 km^2^ or less (Fig. 7). By comparison, 27 of the 59 DD species (45.8%) where it had been possible to approximate their AOO, were recorded at 2,000 km^2^ or less (Fig. 7). Furthermore, many of the 36.8% of DD species that are wider-ranging (AOO >2,000 km^2^) are based on infrequent sightings. It would therefore be reasonable to suppose that a substantial proportion of the DD species are potential candidates for listing as threatened.

DD species are overwhelmingly found in the Indo-Pacific. There are three in the Eastern Atlantic, eight in the Western Atlantic, 75 in the Indo-Pacific and one in the Eastern Pacific. Outside the Indo-Pacific there is just a single species with a known AOO of more than 2,000 km^2^.

## Discussion

It is widely believed that extinction risk in the sea is less likely than in the terrestrial environment and that this is supported by the fossil record [2], [3]. This view is based largely on perceived high fecundity, greater dispersal ability and geographic range size [40]. With 6.5% of *Conus* species at risk globally this would appear to follow this perception, however, in regions offering reduced dispersal opportunity, such as the whole of the Eastern Atlantic, 25.5% of species are threatened. Cone snails here have a similar level of extinction risk to species in well-assessed terrestrial taxa, such as freshwater invertebrates (34% of 7,784 species assessed at risk), lepidoptera (from 8.5% of butterflies in Europe to 17% in the U.S. at risk), European terrestrial molluscs (20% at risk) [41], [42] and bryophyte flora from the Canaries (21% at risk) [43]. Contributing to the pattern seen, many cone snails have limited dispersal ability, small geographic ranges and/or are rare. The level of extinction risk is similar in other well assessed marine taxa, including corals (27% of species at risk) [7], [44] and scombrid and billfish (11% of 61 species at risk) [45]. Given the rapid escalation of threats to the marine environment [46], if the pattern seen in these groups is typical of marine species generally, then there is a high risk that extinctions will soon become common in the sea, just as they now are on land.

Our global assessment of the conservation status of all 632 cone snails shows that three-quarters (75.6%) of species are classified as Least Concern under IUCN Red List standards. However, beneath this relatively optimistic result lies a picture of substantial regional variations with indicators signalling wider concerns. In the Eastern Atlantic along the shores of Senegal, Cape Verde and Angola, species restricted in their range and subject to the effects of industrialisation and urbanisation face an elevated risk of extinction. Endemism for marine species occurs most commonly in isolated island groups where the original dispersal was assisted by a pelagic larval stage or by transport on rafting matter [47]. Endemics may also be found where there may be non-reversing currents transporting water away from the tropics towards higher latitudes [40]. All Cape Verde endemic *Conus* have a non-planktonic larval stage having lost the ability during speciation to feed during larval dispersal [48]. This conforms to the hypothesis that non-planktonic, i.e. lecithotrophic, species of *Conus* commonly originate from planktotrophic species [49]. All three species assessed as Critically Endangered occur in the waters off Cape Verde where they are exposed to habitat degraded through coastal development primarily driven by tourism. Similarly, of the 11 Endangered species, four are found in Cape Verde with the remaining seven occurring off the coast of Senegal, in particular the Dakar peninsula, where high levels of pollution from industrial and residential effluent is thought to be the driver of declining abundance and observable reductions in body size. A further 11 species (40.7%) of the 27 assessed as Vulnerable occur in Cape Verde, Senegal and Angola, making West Africa home to 61% of the 41 *Conus* species threatened with extinction. Of the remaining 16 species categorised as Vulnerable, seven are found in the Western Atlantic where they are primarily exposed to coastal development, tourism and shell collecting. The remaining nine occur in the Indian Ocean where petrochemicals, shell-collecting and elevated sea-surface temperatures represent the principal causes of decline.

### Threats

#### Overfishing

The effect of overfishing on the abundance of fish stocks has been extensively reported in both the scientific and general press over many years [50], [51]. However, threats to invertebrates from fishing are seldom equated with extinction, especially marine molluscs. Although extremely unusual, near-extinctions in this group have occurred in the recent past; for example in the white abalone *Haliotus sorenseni* from southern California and Baja California by the mid-1990s had been fished to the edge of extinction [52]. Once counted in the millions there are now probably less than 1,600 individuals remaining.

Amongst marine molluscs, most species are sought by shell collectors [12]. Although this does not threaten the survival of the vast majority of molluscs, shell collecting has undoubtedly caused the decline and endangerment of some species, particularly ‘trophy’ shells. Throughout the Indo-Pacific, the spectacular giant triton (*Charonia tritonis*), has been extensively fished and in many areas has been extirpated [53]. Similarly, although primarily removed for its adductor muscles, the giant clam (*Tridacna gigas*) the largest of all bivalve molluscs, has met the same fate [54]. In Zanzibar, East Africa, the cowries *Cypraea tigris, C. histrio* and *C. lynx* were found to be up to 18 times less abundant in exploited tourist areas [8]. We identified three rare species of *Conus* threatened by shell collecting: *C. richardbinghami* from the Bahamas and *C. immelmani* and *C. julii* from the Mascarenes. Taxa already facing pressures from factors such as pollution may be pushed further towards extinction by gathering for shells, yet warning indicators such as sudden price inflation on the shell market may not alone warrant inclusion to a threatened category.

#### Bioprospecting

*Conus* is exceptionally important to biomedical science, although there is dispute about the number of animals taken for their bioactive compounds. To protect their intellectual property, pharmaceutical companies are silent on the issue, but researchers are adamant that volumes are negligible. In their dialogue in *Science* Chivian et al. (2003; 2004) raised important concerns about the quantity of cone snails taken from the wild, indicating that thousands were then collected to satisfy research demands [55], [56]. This was forcefully rebutted by Duda et al. (2004) who reviewed recent conotoxin research from which they determined that a maximum of 20 research groups were working on *Conus* toxins at that time, and that any single characterisation required fewer than 21 animals to be sacrificed [57]. Regardless of where the true determinant lies, balancing the legitimate needs of medical research without further compromising natural resources is essential. Fortunately, alternative, more sustainable options are now available including milking venom without killing the animal [58], polymerase chain reaction (PCR) sequencing of DNA fragments that requires just one specimen [59] and more recently digital marine bioprospecting using massive parallel deep sequencing of transcriptomes that requires only minute samples of bioactive material [60].

#### Habitat loss

It has been shown that habit loss leads to declines in species richness, reduced biomass and loss of complexity [61], [62], often accompanied by colonisation by species that inhibit recovery [63]. Virtually all of the world's ‘trawlable’ area of continental shelf has already been altered, and about half the area of all the continental shelves is hit by trawls every year [64], changing the structure and function of habitats, destroying assemblages and resulting in homogenisation of the seabed [65]. Of 133 marine species that have been recorded as having gone extinct either regionally or globally, 37% were attributed entirely or in part to habitat loss [66]. Extinctions of marine gastropod molluscs from loss of habitat are set to continue and include the horn snail *Cerithidea fuscata* from southern California last seen in 1935, the eelgrass limpet *Lottia alveus alveus* from the northwest USA last collected in 1929, and from the 19^th^ century the rocky shore limpet ‘*Colisella*’ *edmitchelli* also from southern California and the periwinkle *Littoraria flam*mea from China; all driven to extinction through loss of habitat from anthropogenic causes, with the possible exception of the eelgrass limpet that lost its habitat from a slime mould that may have been introduced from ships' ballast [2], [34].

Our assessment found that with the exception of three species made vulnerable by shell collecting (see above), all 38 other *Conus* species threatened with extinction are impacted to some degree by habitat loss, either directly from coastal and port development or indirectly from pollution or from human exacerbated natural occurrences such as El Niño/La Niña–Southern Oscillation (ENSO) warm-water events (Fig. 6).

### Red List comparatives

Our *Conus* assessment is the first global study for the IUCN Red List for any marine gastropod mollusc genus and one of the few for marine invertebrates. Other marine invertebrates that have been the subject of a global assessment include 845 reef-building corals, 247 lobsters and 195 cuttlefishes [44]. Data Deficiency is a common thread throughout each of these studies with 17%, 35% and 76% of species for each respective grouping [44] compared to 14% for *Conus*. Preliminary results available for oceanic squid show that 57% of this group are of Least Concern with the remaining 43% Data Deficient. As with the data deficient cone snails, many of these cephalopods are deep-water species that have only been captured on a few occasions [44].

Of the 845 corals that have been globally assessed 27.3% fall into a threatened category with a further 20.8% near threatened, although prior to the massive bleaching event of 1998 it has been estimated that 95.3% of non-DD species would have been categorised as Least Concern [7]. The exceptional ENSO event which resulted in this bleaching largely devalues any post-event comparison, although it has been shown that La Niña can impact some mollusc assemblages through stress, changes in productivity and availability of dietary preferences [67]. In Australia, the La Niña event of 2010-11 gave the highest monthly Southern Oscillation Index values on record accompanied by elevated sea-surface temperatures in Western Australia [68]. In the region around Geraldton to Shark Bay including the Abrolhos Islands, this coincided with an estimated 50% mortality in molluscs that included *Conus compressus* (H. Morrison pers. comm. 2011). Scleractinian corals and molluscs, including *Conus*, also share the threat of ocean acidification with the prospect of arrested development in their aragonite-forming structures [69], [70]. Of all the threats faced by these fauna this is the most intractable and one that could even determine their continued existence.

For freshwater molluscs, Red List assessments have been completed for 1,500 of the 5,000 described species [6], [42]. Results show that out of 7,784 freshwater invertebrates assessed to date, gastropods are the most threatened group with a threat range of 33% (if no DD species are threatened) to 68% (if all DD species are threatened) [42]. In common with *Conus*, the threatened species include range-restricted habitat specialists that are particularly at risk from loss of habitat and pollution.

### Further Research and Conservation Priorities

As a global assessment for conservation has not been undertaken on any other marine gastropod it is not possible to explore relationships between different gastropod genera to identify commonality of risks. Further research is urgently needed to address this issue.

Data Deficient *Conus* species can normally be characterised by minimal sightings and a lack of data on distribution. This may result from their bathymetric profile (39% of DD species occur only at 50 m or more) and/or genuine rarity. However, DD species share a common trait with those categorised as threatened in having a higher percentage with restricted range than those from the general population, with the implication that there could be a significant proportion of DD species at risk. Bearing the title ‘Data Deficient’ or ‘Near Threatened’ places these 113 species jointly away from the spotlight afforded the 41 species in the three threatened categories. However, with the potential to double the number of species at risk it is essential that the taxa that make up these categories should not be ignored but instead benefit from further research into their true status.

One of the primary sources of information on species distribution, habitats, populations and threats for our Red List assessment has been the specimen shell trade. In many parts of the developing world, trade in shells provides valuable additional revenue to some of the poorest families living along tropical coastlines. Research is needed to assess the threat from rare shell collecting towards mollusc population decline to determine what measures should be taken to enable this activity to continue sustainably while at the same time allowing for protection of vulnerable species.

The need to identify conservation strategies for all species at risk is compelling, although for developing nations, snail conservation is unlikely to become a driver for environmental improvements. In the absence of in situ conservation measures, captive breeding programmes may ultimately be necessary, such as those undertaken for tree snails of the genus *Partula* from the Pacific Islands [71]. At present, except possibly for species such a *C. pennaceus* and *C. textile* that emerge as mature veligers, this is not a viable option, as the complexity of plankton essential for developing larvae cannot be easily replicated [28].

Over half (53.6%) of all *Conus* species occur at depths of 5 m or less where they are susceptible to gleaning, and nearly three-quarters (74.5%) occur at or above recreational SCUBA diving depths of 30 m or less. Marine Protected Areas (MPAs) offer one of the few sanctuaries, but regional authorities need encouragement to strengthen enforcement and to erect prominent signage against shell gathering within MPAs.

Cape Verde presents a special case in *Conus* conservation. With 45.3% of its species at risk there is a strong argument for legislating against export of both animals and shells, and with manageable borders the country is ideally suited to export controls. This small archipelago also signals a warning to other nations developing their coastal infrastructure: new roads bring visitors to areas previously protected by their isolation, and illegal sand removal for construction from beaches and shallow water of the littoral zone [72] pose a constant threat to habitat. Regional authorities should be required to undertake environmental impact assessments that take account of these issues when planning new developments.

The toxins that make *Conus* so successful are generally unique to each species [59] and any extinction in the genus could in turn deprive science of a potential pharmacological resource. The extraordinary number of species and the global distribution of these tropical snails make them an important contributor to marine biodiversity, and with the appeal of their shells they help support some of the world's poorest people.

Finally, there exists a well-defined community of cone snail aficionados who together are highly influential in the trade in cone shells. This includes leading academics as well as collectors and dealers. A positive first step from our Red Listing is that following a preliminary presentation of our findings at their international convention, a core of members has been motivated to explore a voluntary embargo in trade of critically endangered species and to consider this also for other *Conus* species at risk [73].
